# Efficacy and safety of fire-needle in the treatment of gouty arthritis: A protocol for systematic review and meta analysis

**DOI:** 10.1097/MD.0000000000021259

**Published:** 2020-07-24

**Authors:** Jiya Sun, Yihua Fan, Zhihua Yang, Rui Jin, Ping Xin, Xuemeng Cai, Xinju Li, Shenjun Wang

**Affiliations:** a Tianjin University of Traditional Chinese Medicine; b National Clinical Research Center for Chinese Medicine Acupuncture and Moxibustion; c First Teaching Hospital of Tianjin University of Traditional Chinese Medicine, Tianjin, P. R. China.

**Keywords:** fire needle, gouty arthritis, protocol, randomized controlled trials, systematic review

## Abstract

**Background:**

Fire needle therapy is an ancient external treatment method of traditional Chinese medicine. This therapy is simple to operate and has fewer side effects. Gouty arthritis (GA) is common disease that is often characterized by high excruciating pain on joint. Evidence from clinical studies show that fire needle exerts therapeutic effects on gout arthritis, but no evidence-based medicine is available. This study aimed to evaluate the efficacy and safety of fire acupuncture in the treatment of gout arthritis.

**Methods:**

Randomized controlled trials of fire needle in the treatment of GA published until May 2020 will be searched in the English databases (PubMed, EMBASE, Web of Science, the Cochrane Library) and Chinese databases (China National Knowledge Infrastructure, the Chongqing VIP Chinese Science and Technology Periodical Database, Wanfang database, and China Biomedical Literature Database). Additional search will be performed on Google academy and Baidu Academy. Data will be extracted from the studies by 2 reviewers working independently. Subsequently, quality assessment and a meta-analysis will be carried out for the studies using RevMan 5.3.

**Results:**

The efficacy and safety of fire needle in the treatment of GA will be evaluated based on overall effective rate, visual analog scale, blood uric acid, C-reactive protein, joint swelling and pain score, adverse reaction rate, and other clinical outcomes.

**Conclusions:**

The proposed systematic review and meta-analysis are expected to provide reliable evidence for the clinical benefits of fire-needle therapy in GA.

## 1 Introduction

Gouty arthritis (GA) is an inflammatory pathologic reaction caused by urate deposition in articular, synovium, cartilage, and other tissues. GA is characterized by sudden severe pain, swelling in 1 or more joints, limited joint movement, and dysfunction.^[1–3]^ Its pathogenesis is linked to purine metabolism disorders which result in the excessive production of uric acid in blood or synovial fluid.^[4]^ Data show that GA is prevalent among older men and postmenopausal women, and it is an important risk factor for cardiovascular and cerebrovascular diseases.^[5]^ Some of the risk factors associated with GA include drinking, infection, and trauma. GA can progress into chronic gout, causing joint deformity, gouty nephropathy, and other life-threatening conditions.^[6–9]^

Acupuncture and moxibustion are traditional therapies used to treat various diseases. As one of the therapies recorded in huangdi neijing, the earliest medical book in China, fire needle enhances immunity and regulates blood circulation. Its main advantages include safety, reliability, and convenience of use.^[10]^

Evidence from randomized controlled trials (RCTs) show that fire needle improves pain and blood uric acid in patients with GA. Moreover, fire needle therapy has high cure rate, and is associated with low recurrence rate, and few adverse reaction.^[11–13]^ However, the use of different protocols and indicators in clinical trials has led to inconsistent results, which are less reliable for effective clinical application of fire needle therapy. In this protocol, we aim to objectively evaluate the efficacy and safety of fire needles in the treatment of gout arthritis, with the aim of providing reliable evidence for the clinical application of fire needles in GA.

## 2 Methods

### 2.1 Protocol register

This protocol of systematic review and meta-analysis has been drafted under the guidance of the preferred reporting items for systematic reviews and meta-analyses. Moreover, it has been registered on open science framework (OSF) on May 17,2020. (Registration number: DOI 10.17605/OSF.IO/7E25Q).

### 2.2 Ethics

Ethical approval is not required because there is no patient recruitment and personal information collection, and the data included in our study are derived from published literature.

### 2.3 Eligibility criteria

#### 2.3.1 Types of studies

We will collect data from available RCTs on fire needle treatment for GA, regardless of blinding, publication status, region, but language will be restricted to Chinese and English.

#### 2.3.2 Types of participants

The patients with GA in the studies should be diagnosed according to the diagnostic criteria and other tests, regardless of nationality, race, gender, age, course of disease, and location of disease.

#### 2.3.3 Types of interventions

Only studies in which the treatment group received fire needle therapy alone or fire needle combined with western medicine will be included. The enrollment of studies will not apply restrictions concerning the operation method used, acupoints and treatment course. Patients in control group will receive western medicine, with no limitation on the type, dosage, and treatment course.

#### 2.3.4 Types of outcome measures

(1) Primary outcome
  For this study, the primary outcome is the overall effective rate.
(2) Secondary outcomes
  Visual analogue scale score, blood uric acid, C-reactive protein, joint swelling and pain score, and incidence of adverse reactions.

### 2.4 Exclusion criteria

Studies will be excluded if the following items are observed:

(1) Repeated publication
(2) Articles published as abstracts and additional data cannot be obtained even after contacting the author
(3) Incomplete data or research with obvious errors
(4) Randomized or distributive concealment assessed as high bias risk.^[14]^

### 2.5 Search methods for study identification

According to the preferred reporting items for systematic reviews and meta-analyses, “fire needle”, “fire-needle”, “gout” will be used as search terms to identify eligible studies in the following databases from their inception to May 2020: PubMed, Embase, Web of Science, the Cochrane Library, China National Knowledge Infrastructure, the Chongqing VIP Chinese Science and Technology Periodical Database, Wanfang database, and China Biomedical Literature Database. The same strategy was used to manually search Baidu academic and Google academic. The search strategy performed for the PubMed is shown in Table 1 Search strategy in PubMed database.

**Table 1:**
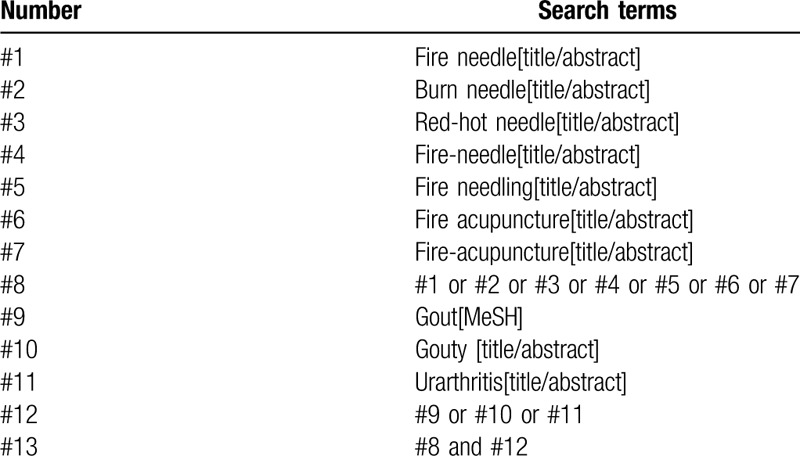
PubMed search strategy.

### 2.6 Data extraction and management

**Figure 1:**
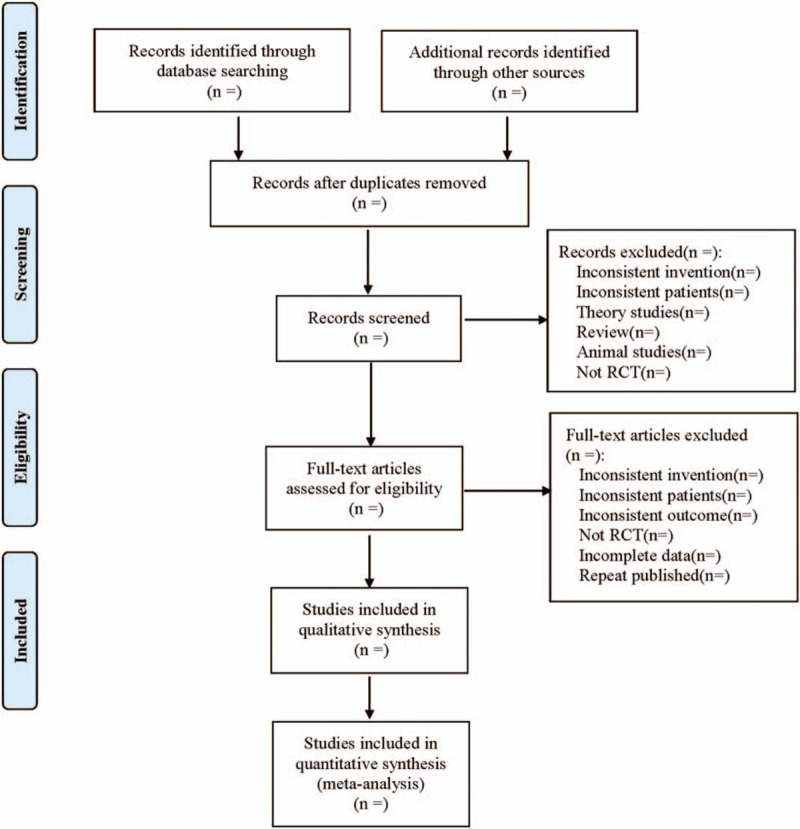
Flow diagram of study selection process.

Two researchers will retrieve documents from the databases mentioned above using Endnote X7 in conformity with the research selection in the Cochrane collaborative network system evaluator's manual version 5.0, the PRISMA flow chart. Two researchers will independently select documents by reading the title and abstract. Studies deemed relevant will be preliminarily selected. Next, another round of selection will be conducted via reading the full text of each document while referring to the inclusion and exclusion criteria. Finally, the papers will be cross-checked by the 2 investigators. Any discrepancies observed were resolved through consensus by discussing with a third party. The following details will be extracted: the first author, the year of publication, the basic information of the study (participant details, the interventions and the outcome, course of treatment, and assessment criteria, the outcomes). In cases where some information is lacking, the authors of the studies will be reached out. The screening process is shown in systematic reviews and meta-analyses flow chart (Fig. 1 Flow diagram of study selection process).

### 2.7 Literature quality evaluation

The Cochrane Collaboration's tool for assessing risk of bias will be used to assess the risk of bias among studies. The 2 researchers will assess the studies as low-risk, unclear and high-risk items one by one. After completion of each step, all data will be cross-checked, differences will be discussed, and the third researcher will be involved if no agreement is reached.

### 2.8 Statistical analysis

This meta-analysis will be performed using statistical software (Review Manage software [RevMan 5.3]). For continuous variables, the weighted mean difference will be used to evaluate the extracted data. For dichotomous variables, thestandard mean difference will be used as the effect quantity, all of which expressed by 95% confidence interval. If *P* ≥ .1, I^*2*^ ≤ 50% indicates low heterogeneity, fixed effect model will be used for meta-analysis. If *P* < .1, I^*2*^ > 50% are obtained, heterogeneity will be considered significant between studies, and the sources of heterogeneity will be analyzed. Clinical heterogeneity will be treated by subgroup analysis. In the absence of significant clinical heterogeneity and methodological heterogeneity, statistical heterogeneity will be considered, and the random effect model will be used for analysis. If clinical heterogeneity is significantly high for subgroup analysis, meta-analysis will not be carried out, only descriptive analysis will be carried out.

#### 2.8.1 Dealing with missing data

In case of missing data, the author will be contacted via email to obtain the information. If the author cannot be contacted, or the author cannot provide relevant data, descriptive analysis will be conducted instead of meta-analysis.

#### 2.8.2 Subgroup analysis

The treatment group receiving simple fire needle and fire needle combined with western medicine will be used to perform subgroup analysis. Based on age, participants will be divided into young and elderly patients subgroups. Subgroup analysis will be performed according to the body part affected by GA and the course of treatment.

#### 2.8.3 Sensitivity analysis

In order to judge the stability of outcome indicators, sensitivity analysis was used to analyze each outcome indicator.

#### 2.8.4 Assessment of reporting biases

If the number of studies included in a certain outcome index is no less than 10, funnel chart is used to evaluate publication bias. In addition, Egger and Begg test were used for the evaluation of potential publication bias.

#### 2.8.5 Summary of evidence

The evidence evaluation of results will be summarized using GRADE method. The quality of evidence is divided into high, medium, low, and pretty low quality.

## 3 Discussion

GA belongs to “li jie”, “bi zheng” category of the traditional Chinese medicine, which includes conditions such as multifactor insufficiency, daily life disorder, eating, and induce. It limits the patient's daily activities,^[15]^ and seriously deteriorates the quality of life of patients.^[16]^ Evidence indicates that the incidence of GA has been on the rise in recent years even in younger patients.^[17,18]^ Modern medicine used to treat GA are designed to inhibit uric acid production, promote uric acid excretion and prevent inflammation thereby relieving acute symptoms. However, they are associated with side effects of different degrees, with poor long-term effects.^[19]^ This calls for alternative treatments for patients with gout arthritis.

Acupuncture and moxibustion therapy are used as complementary therapy in China and abroad for its effectiveness, simplicity and fewer adverse reactions. Other modifications of acupuncture methods, such as percutaneous electrical stimulation^[20]^ and electroacupuncture^[21]^ have been established. The main indication for acupuncture and moxibustion is pain. Fire needle is a traditional acupuncture therapy used to warm meridians and unblock collaterals, remove wind and dampness, promote blood circulation and remove blood stasis, soften and strengthen the body, dispersing the knots, detumescence, and relieve pain.^[22]^ Its WenTong effect is stronger than that of ordinary filiform needle. It is widely used to treat rheumatism bi disease, nervous system diseases, skin diseases, surgical disease, digestive system disease, diabetes, obesity, among other diseases.^[23]^ It is easy to operate, provides rapid treatment and does not retain the needle.

In recent years, fire needle has exhibited good curative effect in patients with GA. In most studies, the Ashi point is used reach the quasi position of rapid needling, the depth of needling is 0.3 to 1.0 inch, 3 to 4 needling. Other studies supplement this therapy with bloodletting therapy. Studies have shown that fire needle stimulates the disease site and reflex point thereby eliminating or improving pathological changes such as local tissue edema, hyperemia, exudation, and adhesion. This speeds up blood circulation and metabolism, and suppresses inflammation.^[24]^ However, the clinical effect of this therapy has not been recognized by international authoritative medical organizations. Therefore, it is necessary to analyze the existing RCT and objectively evaluate the clinical efficacy and safety of fire-needle therapy for GA. In this way, evidence supporting the clinical benefits of fire-needle therapy in GA will be revealed. The proposed study will have some limitations. Due to the special operation techniques in the use of fire needle, it is difficult to apply blind method, and the overall literature quality is not high. Moreover, only studies published in English and Chinese literature will be captured, hence other important research or reports published in other languages may be missed.

## Author contributions

**Data collection:** Zhihua Yang and Rui Jin.

**Funding acquisition:** Xinju Li and Shenjun Wang.

**Resources:** Zhihua Yang and Ping Xin.

**Software:** Jiya Sun.

**Supervision:** Xinju Li and Shenjun Wang.

**Writing – original draft:** Jiya Sun and Yihua Fan.

**Writing – review & editing:** Jiya Sun and Xinju Li.
